# Molecular and culture-based assessment of the microbiome in a zebrafish (*Danio rerio*) housing system during set-up and equilibration

**DOI:** 10.1186/s42523-021-00116-1

**Published:** 2021-08-05

**Authors:** Aaron C. Ericsson, Susheel B. Busi, Daniel J. Davis, Henda Nabli, David C. Eckhoff, Rebecca A. Dorfmeyer, Giedre Turner, Payton S. Oswalt, Marcus J. Crim, Elizabeth C. Bryda

**Affiliations:** 1 Department of Veterinary Pathobiology, College of Veterinary Medicine, University of Missouri, Columbia, MO USA; 2 University of Missouri Metagenomics Center, Columbia, MO USA; 3 Systems Ecology Group, Luxembourg Centre for Systems Biomedicine, University of Luxembourg, Esch-sur-Alzette, Luxembourg; 4 Animal Modeling Core, University of Missouri, Columbia, MO USA; 5 IDEXX BioAnalytics, Columbia, MO USA

**Keywords:** Zebrafish, *Danio rerio*, Aquarium, Recirculating aquaculture system, Housing, Microbiota, Microbiome, Culture

## Abstract

**Background:**

Zebrafish used in research settings are often housed in recirculating aquaculture systems (RAS) which rely on the system microbiome, typically enriched in a biofiltration substrate, to remove the harmful ammonia generated by fish via oxidation. Commercial RAS must be allowed to equilibrate following installation, before fish can be introduced. There is little information available regarding the bacterial community structure in commercial zebrafish housing systems, or the time-point at which the system or biofilter reaches a microbiological equilibrium in RAS in general.

**Methods:**

A zebrafish housing system was monitored at multiple different system sites including tank water in six different tanks, pre- and post-particulate filter water, the fluidized bed biofilter substrate, post-carbon filter water, and water leaving the ultra-violet (UV) disinfection unit and entering the tanks. All of these samples were collected in quadruplicate, from prior to population of the system with zebrafish through 18 weeks post-population, and analyzed using both 16S rRNA amplicon sequencing and culture using multiple agars and annotation of isolates via matrix-assisted laser desorption/ionization-time-of-flight (MALDI-TOF) mass spectrometry. Sequencing data were analyzed using traditional methods, network analyses of longitudinal data, and integration of culture and sequence data.

**Results:**

The water microbiome, dominated by *Cutibacterium* and *Staphylococcus* spp., reached a relatively stable richness and composition by approximately three to four weeks post-population, but continued to evolve in composition throughout the study duration. The microbiomes of the fluidized bed biofilter and water leaving the UV disinfection unit were distinct from water at all other sites. Core taxa detected using molecular methods comprised 36 amplicon sequence variants, 15 of which represented *Proteobacteria* including multiple members of the families *Burkholderiaceae* and *Sphingomonadaceae*. Culture-based screening yielded 36 distinct isolates, and showed moderate agreement with sequencing data.

**Conclusions:**

The microbiome of commercial RAS used for research zebrafish reaches a relatively stable state by four weeks post-population and would be expected to be suitable for experimental use following that time-point.

**Supplementary Information:**

The online version contains supplementary material available at 10.1186/s42523-021-00116-1.

## Background

The laboratory zebrafish (*Danio rerio*) are an attractive model species in biomedical and developmental research, owing to a unique combination of qualities that are important for animal model selection including biological characteristics that can be exploited, the availability of advanced imaging and molecular techniques, and financial feasibility [1]. From a biological perspective, zebrafish share many of the same organ systems as higher vertebrates and, in some situations, may represent the optimal model species for translational research [2, 3]. Moreover, the genetic tractability of zebrafish has allowed the targeted testing of gene function as is performed in knock-out and transgenic rodents [4]. From a purely logistical perspective, zebrafish provide several conveniences such as a reduced regulatory burden compared to research rodents, reduced costs associated with housing and husbandry, and the ability to increase throughput in large-scale surveys of compounds.

Zebrafish are also increasingly used in behavioral and neuropsychological research, both in academia and the industrial sector during the pursuit and development of investigational new drugs (INDs) [5, 6]. With comprehensive and well-characterized behavioral ethograms [7, 8], the effects of various stimuli can be assessed [9–12]. Recent reviews highlight the diversity of behavioral traits and social deficits that have been investigated using both adult and larval zebrafish models [13–15].

During the last decade, there has been a growing appreciation of the influence of the gut microbiota (GM) on human and animal health, and in animal models. Specifically, the GM has been identified as a potential source of poor experimental reproducibility in animal models [16], while also providing discoveries regarding the mechanisms by which the GM influences host development and health, both physical and mental [17, 18]. The fact that zebrafish are often housed in aquaria filled with a recirculated filtered water supply brings into question the nature of bacterial populations present at different levels of commercial zebrafish housing systems. Moreover, the establishment of environmental bacterial populations in a recirculating aquaculture system (RAS) is essential to accommodate the removal of nitrogenous waste (i.e., ammonia), the accumulation of which is highly toxic to fish [19, 20]. While it is recommended to allow new zebrafish housing systems to equilibrate for one to two weeks prior to population, and to populate any aquaculture system gradually [21], there are minimal empirical data documenting the time-course at which the microbiological communities within commercial RAS reach a steady state. As the environmental microbiota may influence zebrafish physiology or host-associated microbiota [22], this information is important to ensure robust, reproducible data from zebrafish experiments.

In recent years, there has been an increasing awareness of a reproducibility crisis affecting in vivo experiments. As in other model organisms, lack of reproducibility in zebrafish experiments can be caused by differences in intrinsic factors, such as background genetics [23], or extrinsic factors, which include diet, housing systems and various other aspects of the environment [24]. Environmental microbiota impact human microbiomes and human health [25]. Thus, it is likely that environmental microbiota impact zebrafish microbiomes and zebrafish health, and by extension, experimental reproducibility. To date, the focus on the environment from a reproducibility perspective has been limited to the impact of abiotic factors such as water quality, environmental enrichment, structural complexity, and social factors such as stocking density [26].

The objectives of the current study were therefore to characterize the composition and core taxa present throughout a commercial zebrafish housing system during set-up and population, to characterize the microbial interaction networks during this initial period within the individual system sites and as a whole, and to identify the culturable portion of the system during that time. We therefore sampled six different sites of a new zebrafish housing system at twelve weekly or biweekly time-points, beginning immediately prior to population of several tanks with adult zebrafish (one week post-installation and introduction of circulating water) and ending at 18 weeks post-population. Samples were then subjected to 16S rRNA amplicon sequencing and a culture-based survey of bacterial communities in each system site throughout the study period.

## Methods

### Experimental design and sample collection

**Fig. 1:**
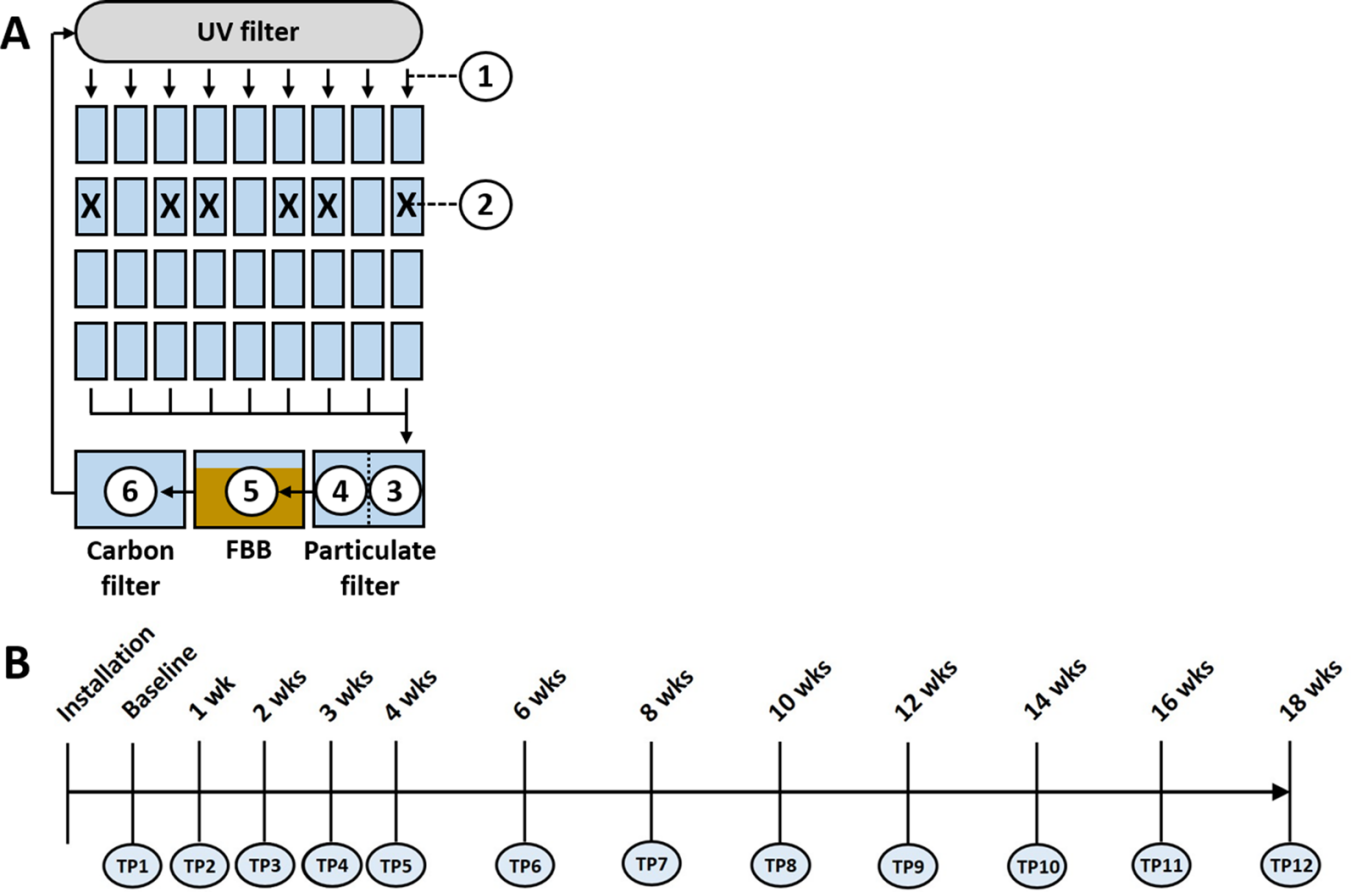
Schematic diagram of the recirculating system, and the sampled sites including post-UV disinfection water (1), tank water from six different tanks in which fish were introduced following collection of baseline samples, denoted by X (2), pre-particulate filter water (3), post-particulate filter water (4), the fluidized bed biofilter (FBB) (5), and carbon filter water (6) (**A**), and timeline showing the time-points (TP) at which samples were collected from each site, in quadruplicate (**B**)

The housing system used in the current study was a ZS560 system from Aquaneering, Incorporated (San Diego, CA), equipped with a 6 GPM self-contained filtration system including a reusable solid particle filter, a fluidized bed biological filter, dual carbon filters, and a standard 40-W ultraviolet disinfection unit. Following commercial installation and set-up of the system, deionized (DI) water was introduced and the system was allowed to circulate unpopulated for one week. Initial baseline samples were then collected at time-point 1 (TP1), immediately prior to population of six tanks (labeled A through F) with 6 to 11 adult zebrafish per tank. Samples (1 mL of water or substrate) were collected from six different sites on the system including 1) post-UV disinfection prior to entering the housing tanks, 2) housing tanks, 3) pre-particulate filtration, 4) post-particulate filtration, 5) the fluidized bed biofilter (FBB), and 6) post-Carbon filtration (Fig. 1). Additionally, tank water samples were collected from three previously established tanks from a housing system constructed according to the design of Kim et al. [27], that had housed zebrafish used to populate the new system (three tanks, four replicates/tank). Subsequent samples were then collected from the six aforementioned sites weekly for one month (TP1 through TP4), and then every two weeks for the following three months (TP5 through TP12). Four technical replicates were collected at each site and time-point; additionally, six housing tanks were sampled at each time-point (with four technical replicates per tank).

### Animals and husbandry

All zebrafish were maintained in an AAALAC International-accredited facility at the University of Missouri (Columbia, MO) in accordance with the guidelines presented in the Guide for the Care and Use of Laboratory Animals [28]. All husbandry procedures were approved by the University of Missouri Animal Care and Use Committee. The zebrafish were 61 adult, mixed-sex, wild-type fish originally obtained from Aquatica BioTech (Sun City Center, FL). Prior to stocking on the ZS560 system (Aquaneering, Incorporated), zebrafish were housed on an established RAS assembled in-housed based on a previously published design [27]. Zebrafish were maintained on a 14:10-h light:dark cycle at 27 °C. Water quality was tested weekly using Lifegard Test Strips Aquatics 6 way All Purpose Test Kit per the manufacturer instructions (Aquaneering Inc., catalog number STK6), with the following limits of detection: nitrite, 0 to 10 ppm (mg/L); nitrate, 0 to 200 ppm; total hardness, 0 to 300 ppm; total alkalinity, 0 to 300 ppm; and pH, 6.2 to 8.4. Water quality parameters were maintained as follows: pH, 8.08 (at start-up)–7.27; total ammonia nitrogen, 0 ppm; nitrite, 0 ppm; nitrate < 20 ppm; alkalinity 40–80 ppm; and hardness, ∼80 ppm. System pH was regulated by the addition of salt and sodium bicarbonate to water via an automatic dosing system. Zebrafish were fed once daily with a commercially available feed (TetraMin Plus tropical flake food).

### DNA extraction

DNA was extracted from 750 µL of each sample (with no prior filtration or concentration) using QIAamp® DNA PowerFecal® kits (Qiagen®), according to the manufacturer’s instructions with the exception that, rather than performing the initial homogenization of samples using the vortex adapter described in the protocol, samples were homogenized in the provided bead tubes using a TissueLyser II (Qiagen®) for three minutes at 30 Hz/sec, before proceeding according to the protocol and eluting in 100 µL of elution buffer (Qiagen®). DNA yields were quantified via fluorometry (Qubit® 2.0, Invitrogen™) using quant-iT™ BR dsDNA reagent kits (Thermo Fisher Scientific). Routine negative controls consisting of unused reagents reproducibly yield between 0 and 100 sequences. Positive controls for DNA extraction and sequencing consisted of mock community standards containing 10 different microbial taxa (ZymoBIOMICS™, D6300), all of which were detected in the resulting sequencing data with zero contaminating sequences.

### 16S rRNA library preparation and sequencing

Extracted DNA was processed at the University of Missouri DNA Core Facility (Columbia, MO). Bacterial 16S rRNA amplicon libraries were generated via amplification of the V4 region of the 16S rRNA gene with universal primers (U515F/806R), flanked by Illumina® standard adapter sequences [29, 30]. Primers used for amplification used the TruSeq DUI adapter design, and dual-indexed forward and reverse primers were used in all reactions. PCR was performed in 50 µL reactions containing all available metagenomic DNA concentrated to a uniform volume, primers (0.2 µM each, IDT), dNTPs (200 µM each, NEB), and Phusion™ high-fidelity DNA polymerase (1U, Thermo Fisher Scientific). Amplification parameters were 98 °C^(3 min)^ + [98 °C^(15 s)^ + 50 °C^(30 s)^ + 72 °C^(30 s)^] × 25 cycles + 72 °C^(7 min)^. Amplicon pools (5 µL/reaction) were combined, thoroughly mixed, and then purified by addition of Axygen® Axyprep Mag™ PCR clean-up beads (Thermo Fisher Scientific) to an equal volume of 50 µL of amplicons and incubated for 15 min at room temperature (RT). Products were washed multiple times with 80% ethanol and the dried pellet resuspended in 32.5 µL EB buffer (Qiagen®), incubated for two minutes at RT, and then placed on the magnetic stand for five minutes. The final amplicon pool was evaluated using the Advanced Analytical Fragment Analyzer automated electrophoresis system, quantified using quant-iT™ HS dsDNA reagent kits (Thermo Fisher Scientific), and diluted according to Illumina’s standard protocol for sequencing on the MiSeq™ instrument using a 2 × 250 bp paired-end read flow cell.

### Bioinformatics

All bioinformatics were performed at the MU Informatics Research Core Facility (Columbia, MO). Cutadapt (version 2.6; https://github.com/marcelm/cutadapt) was used to remove the primer from the 5' end of the forward read. If found, the reverse complement of the primer to the reverse read was then removed from the forward read as were all bases downstream. Thus, a forward read could be trimmed at both ends if the insert was shorter than the amplicon length. The same approach was used on the reverse read, but with the primers in the opposite roles. Read pairs were rejected if one read or the other did not match a 5' primer, and an error-rate of 0.1 was allowed. Two passes were made over each read to ensure removal of the second primer. A minimal overlap of 3 bp with the 3' end of the primer sequence was required for removal.

The QIIME2 [31] DADA2 [32] plugin (version 1.10.0) was used to denoise, de-replicate, and count amplicon sequence variants (ASVs), incorporating the following parameters: 1) forward and reverse reads were truncated to 150 bases, 2) forward and reverse reads with number of expected errors higher than 2.0 were discarded, and 3) Chimeras were detected using the "consensus" method and removed. R version 3.5.1 [33] and Biom version 2.1.7 were used in QIIME2™. Taxonomies were assigned to final sequences using the Silva.v132 database, using the classify-sklearn procedure.

Total ASV count data obtained via QIIME2 were used to determine detected richness and alpha-diversity, using Past3 software [34]. Rarefaction to a uniform sequence count was not performed due to the low coverage of many samples, lack of a clear threshold in coverage, the fact that low coverage was expected a priori due to low biomass, and multiple reports that rarefaction is neither necessary or advisable in most cases [35, 36]. ASV count data were also used to identify the core taxa at different time-points, i.e., early, mid and late, based on the definitions proposed by Risely [37] and the phyloseq R package [38], designed to be a conservative measure preventing noise and spurious ASVs from being identified as being part of the core. Taxa with a minimum relative abundance of 0.1% that were prevalent in at least 50% of all the samples within each grouping at the different time-points were identified to form the core using the *microbiome* R package [39]. Additionally, the *ggAlluvial* package [40] was used to generate plots of taxa found to be overlapping between time-points and within each of the sites. The ASV count information was also used to generate co-occurrence networks within each site, across all time-points, i.e., TP1 through TP12. The log-transformed ASV abundance tables, obtained after processing the 16S rRNA sequences using the DADA2 pipeline implemented into QIIME2, were filtered to remove ASVs with relative abundance greater than or equal to 0.01% of the total number of reads. This level of filtering as based on reports indicating that rare taxa appear in only 1–5% of samples in a metabarcoding dataset [36] and the few, if any, rare taxa are over-represented in microbial networks [41]. Importantly, rare ASVs were removed from network analyses due to their potential to be sequencing artefacts [42] and to reduce false positive results [43]. However, considering a recent report suggesting that low abundance taxa may still influence compositional changes in gut microbiota [44], ASVs below a medium relative abundance of 0.01% were removed to account for any possible role of such taxa in community assembly and composition. Subsequently a weighted conditional-dependence network connecting the ASVs was built using the graphical lasso regression method employed within the Sparse InversE Covariance estimation for Ecological Association and Statistical Inference (Spiec-Easi) package [45]. The graphical lasso method was chosen over a range of independence screening with a nlambda of 30. Manual curation including the removal of self-loops, singleton nodes, and negative edges was performed, followed by generation of network plots using the *igraph* package [46]. Using this package, the nodes were colored according to their respective taxonomical classifications, while the size of the nodes was adjusted to indicate those with highest degree, centrality, and betweenness. The edges in the network graphs were weighted based on the relative abundance and indicate the correlation between two nodes, i.e., taxa.

### Bacterial culture and identification

Replicate water samples from each site were submitted to the microbiology laboratory at IDEXX BioAnalytics (Columbia, MO) and cultured individually for bacterial growth, except for tank water samples, which were collected from three of the six tanks on the Aquaneering rack sampled for 16S rRNA sequencing and pooled as a single sample for each timepoint. Pooling was accomplished by combining 200 µL from the vortexed water samples from each site into a 1.5 mL sterile microcentrifuge tube. For each water sample, sterile PBS was used to prepare three serial dilutions (1:10, 1:100, and 1:1000). A 100 µL inoculum of each undiluted water sample and 100 µL of each dilution were plated separately onto the following bacterial culture media: BBL™ Trypticase™ Soy Agar with 5% sheep blood (TSA II™; Becton Dickinson), BBL™ CDC 5% Sheep Blood Agar with Phenylethyl Alcohol (PEA; Becton Dickinson), Difco™ Xylose Lysine Deoxycholate Agar (XLD; Becton Dickinson), and Tryptone Yeast Extract Salts (TYES) Agar, which was prepared in-house according to a published formulation. [47] Culture plates were incubated aerobically for 5 days at 22 °C. Morphologically unique colony types were selected and harvested from each plate for proteomic analysis using a direct transfer method as previously described. [48] Transferred bacteria were overlaid with 1 µL of a saturated matrix solution of α-cyano-4-hydroxycinnamic acid in 50% acetonitrile and 2.5% trifluoroacetic acid (HCCA, Bruker Daltronics, Billerica, MA) and analyzed by matrix-assisted laser desorption/ionization time-of-flight mass spectrometry (MALDI-TOF MS) using a mass spectrometer (Microflex™, Bruker Daltronics) and flexControl™ software (Bruker Daltronics). Bacterial identification was achieved using automated analysis by MALDI BioTyper® software (Bruker Daltronics) by comparing the collected spectra with integrated reference spectral databases.

### Statistics

Univariate outcome measures related to sample coverage, richness, and α-diversity were compared using a two-way analysis of variance (ANOVA) in a general linear model, with site and time-point as variables, and implemented in SigmaPlot® 14.0. Differences in β-diversity were tested using permutational multivariate ANOVA (PERMANOVA), with site and time-point as variables, and implemented in Past 3.26 [34]. Differences in β-diversity and dispersion were confirmed using the adonis function from the Vegan package in R, with site and time-point as sequential variables. Comparisons were made using both weighted (Bray–Curtis) and unweighted (Jaccard) similarities.

## Results

### Sequencing coverage, richness, and α-diversity plateau at four weeks post-population

Samples returned anywhere from 2 to almost 2 million high-quality sequences. Only three samples were removed from further analysis due to low coverage, leaving a total of 537 samples in the study. Several samples from the initial time-point yielded low read counts, however coverage gradually increased at subsequent time-points, most notably in the Fluidized Bed Biofilter (FBB) and Post-UV disinfection samples, until reaching a peak or plateau at approximately TP5 (four weeks post-population) (Additional file 1). Two-way ANOVA revealed significant main effects of time (*p* < 0.001, F = 26.4) and sample site (*p* < 0.001, F = 288.0) with significant interactions between factors (*p* < 0.001, F = 23.2) reflecting that fact that, within sample site, significant time-dependent differences were detected only in FBB samples.

**Fig. 2:**
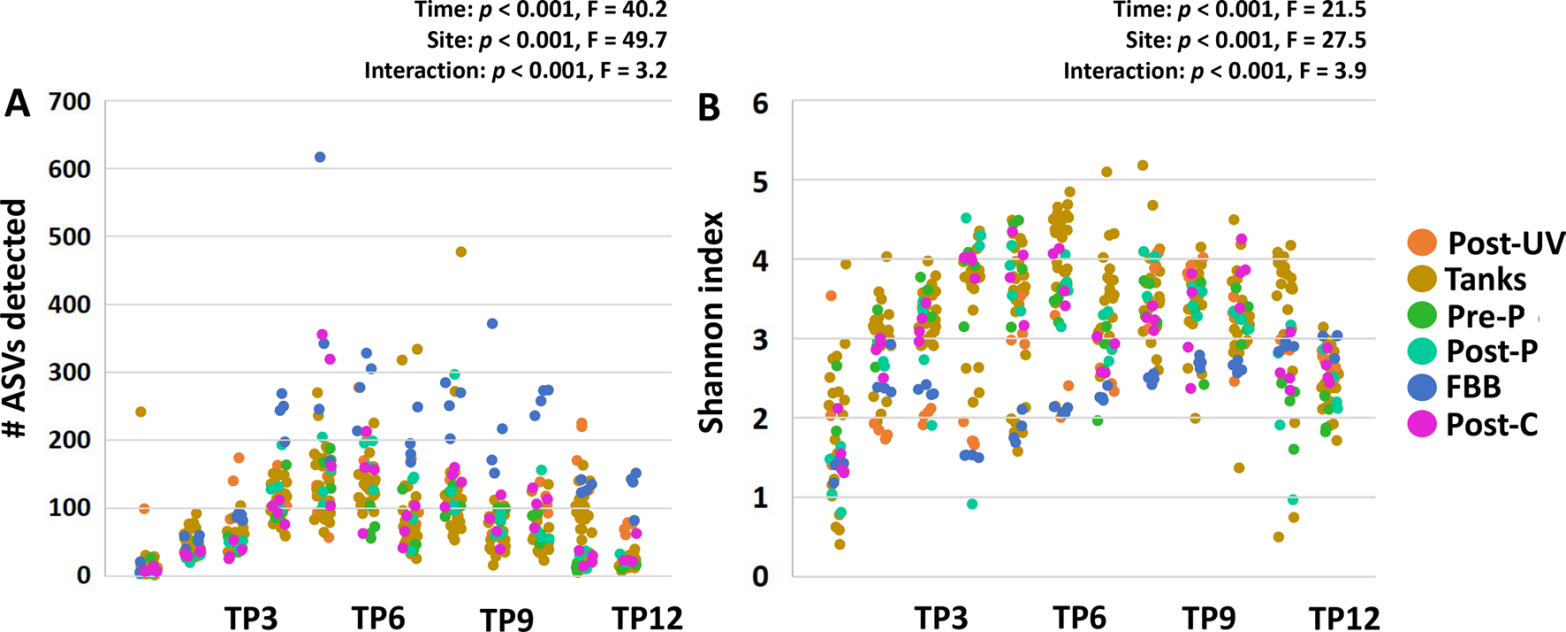
Richness and diversity of the system microbiome plateaus by approximately four weeks post-population. Dot plots showing the detected richness (**A**) and Shannon diversity (**B**) at each sample site (legend at right), and at each time-point (TP) from TP1 through TP12. *p* and F values associated with main effects of time and sample site based on two-way analysis of variance (ANOVA)

Similarly, there were significant effects of time (*p* < 0.001, F = 40.2) and sample site (*p* < 0.001, F = 49.7) on detected richness, which increased gradually in all sample sites between the initial sample and TP4 before reaching a peak or plateau, with the FBB harboring a significantly richer microbiota than other sites at TP4 and all subsequent time-points (p < 0.001, two-way ANOVA) (Fig. 2A). Pairwise comparisons of richness between time-points found significant differences between each of the first four time-points and later time-points, but no significant differences in richness were detected between time-points beyond T4. Likewise, comparison of Shannon diversity detected similar time-dependent (*p* < 0.001; F = 21.5) and site-dependent (*p* < 0.001, F = 27.5) differences, and demonstrated a similar pattern among most sites, with the exception of the FBB which experienced a brief decline at TP4, followed by a slow and gradual increase over the remainder of the study duration (Fig. 2B). Pairwise comparisons of Shannon diversity found significant differences between the first three time-points and later time-points, but no pairwise differences in Shannon diversity beyond T3.

### Dominant taxa established in tanks at four to six weeks post-population

**Fig. 3:**
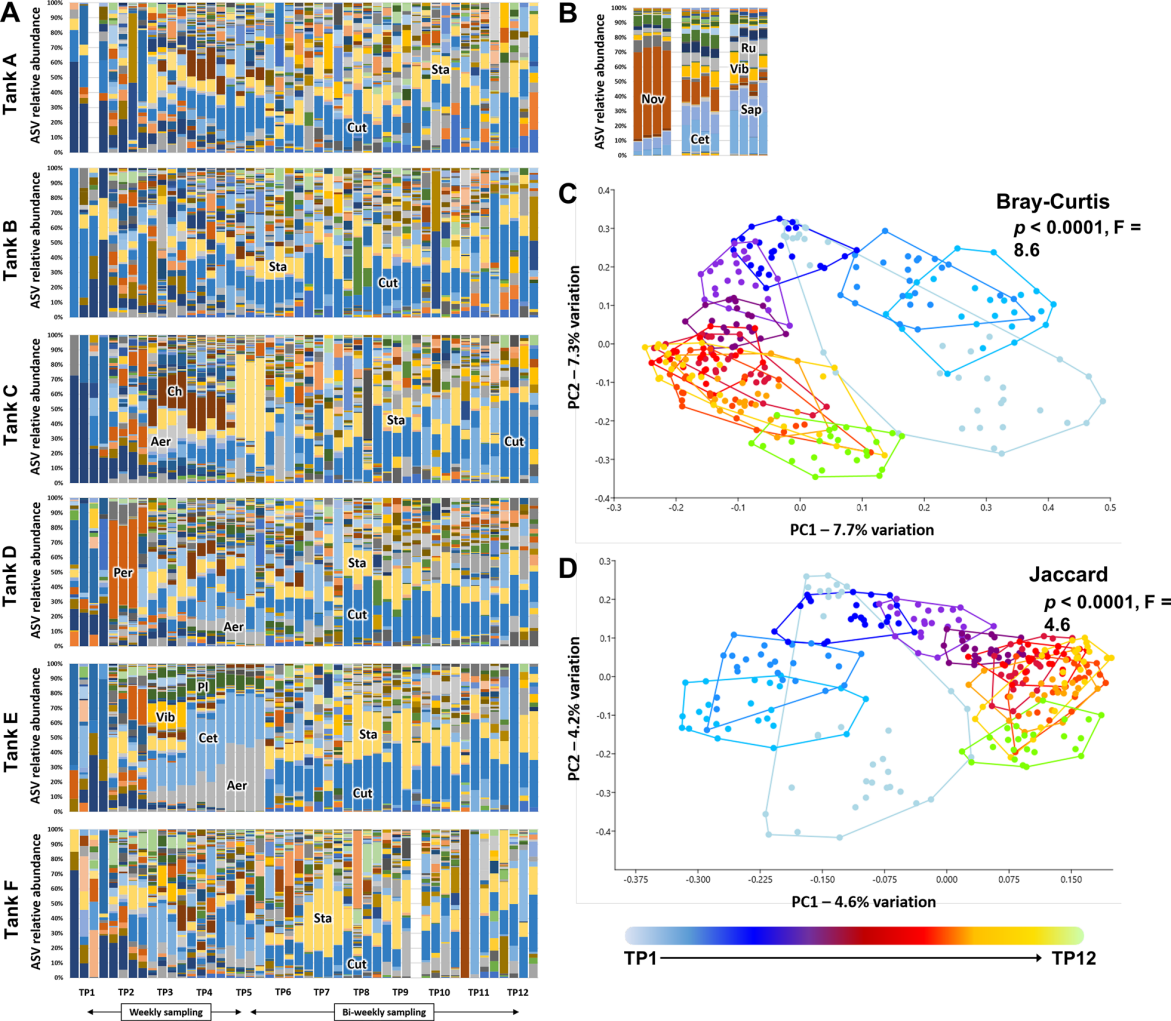
Dominant taxa in tank water are established by two to three weeks post-population. Stacked bar charts showing the progression in microbiome composition in six tanks (A through F), sampled at 12 time-points (4 replicates/time-point) (**A**) and the pre-existing tanks from which fish originated; ASV = amplicon sequence variant, Aer = *Aeromonas*, Cet = *Cetobacterium*, Ch = Chitinophagales, Cut = *Cutibacterium*, Nov = *Novosphigobium*, Per = *Perlucidibaca*, Pl = *Plesiomonas*, Ru = *Runella*, Sap = *Saprospiraceae*, Sta = *Staphylococcus*, Vib = *Vibrio* (**B**). Principal coordinate analysis plots based on Bray–Curtis (**C**) and Jaccard (**D**) similarities. TP = time-point, color-bar legend at bottom; taxonomic abbreviations listed at beginning of manuscript. *p* and F values associated with main effect of time based on one-way permutation multivariate analysis of variance (PERMANOVA)

The composition of the water microbiome in the tanks showed substantial inter-tank variability during the early time-points with transient proliferation of specific taxa unique to each tank (e.g., unresolved members of the order Chitinophagales (Ch) in Tank C, *Perlucidibaca* (Per) in Tank D or *Aeromonas* (Aer) in Tank E), before ending in a uniform dominance across all tanks of two ASVs representing *Staphylococcus* sp. and *Cutibacterium* sp. by TP4 to TP6 (Fig. 3A). This was in contrast to the tank water microbiota found in the pre-existing tanks housing the fish used to populate the new system, dominated by *Cetobacterium* (Cet), *Novosphingobium* (Nov), *Vibrio* (Vib), and *Runella* (Run) spp. (Fig. 3B). While many of those taxa were detected in the new system (primarily within the FBB), they did not achieve the levels seen in the pre-existing tanks. PCoA plots revealed a gradual time-dependent change in tank water microbiome structure (*p* < 0.0001, F = 8.6, Fig. 3C) and composition (*p* < 0.0001, F = 4.6, Fig. 3D), which slowed by TP7 (eight weeks post-population) but continued to shift throughout the entire study duration.

### FBB and post-UV disinfection water harbor distinct microbiomes

**Fig. 4:**
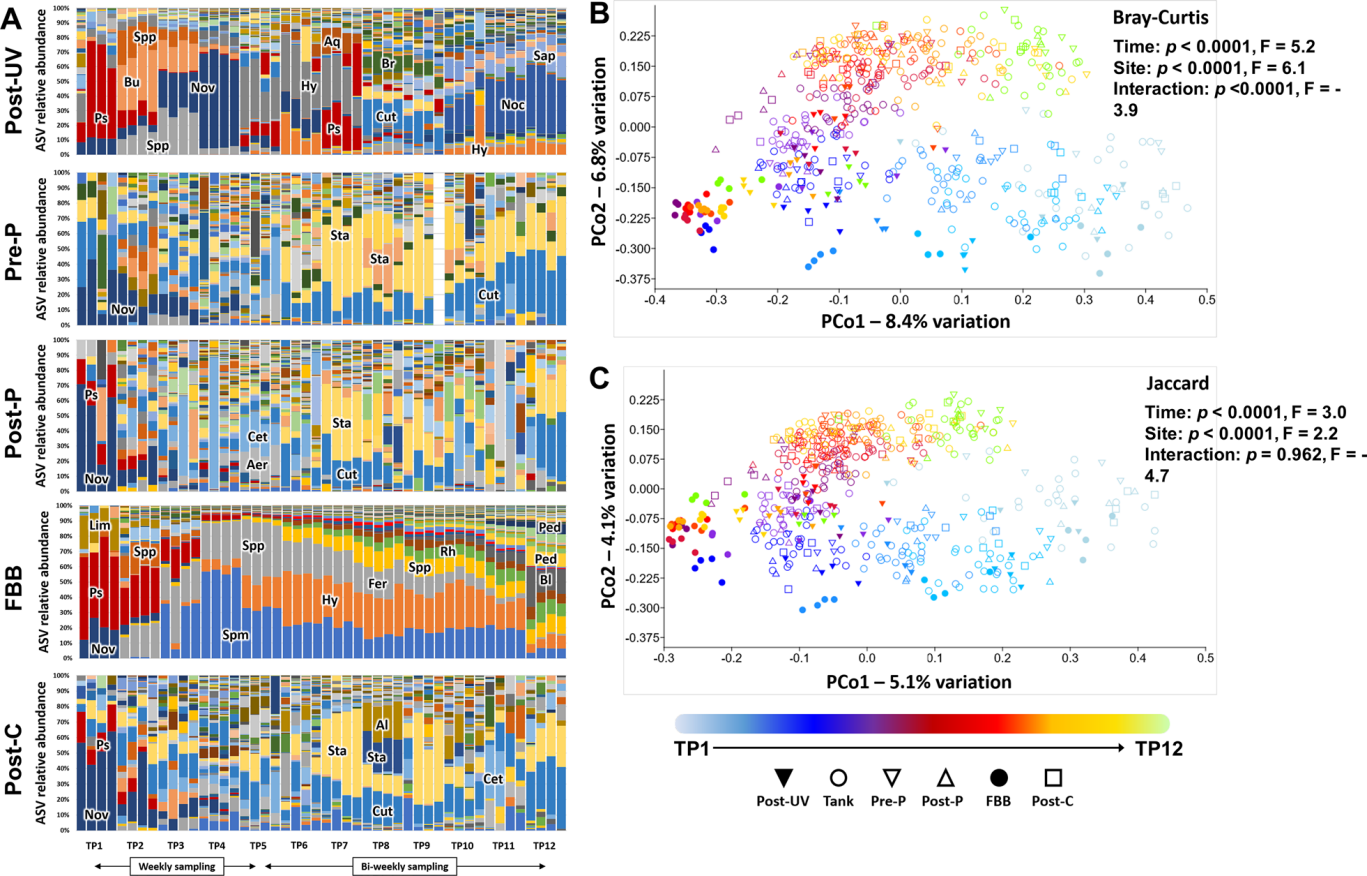
Post-UV disinfection water and FBB harbor dynamic communities, distinct from other sites. Stacked bar charts showing the progression in microbiome composition in the post-UV disinfection water entering the tanks (Post-UV disinfection), the pre- and post-particulate filter water (Pre-P and Post-P, respectively), the fluidized bed biofilter (FBB), and post-carbon filter water (Post-C), sampled at 12 time-points (4 replicates/time-point); ASV = amplicon sequence variant, Aer = *Aeromonas*, Al = *Alloiococcus*, Aq = *Aquabacterium*, Bl = *Blastocatellaceae*, Br = *Bradyrhizobium*, Bu = *Burkholderiaceae*, Cet = *Cetobacterium*, Ch = *Chitinophagales*, Cut = *Cutibacterium*, FBB = fluidized bed biofilter, Fer = *Ferruginibacter*, Hy = *Hydrogenophaga*, Lim = *Limnobacter*, Noc = *Nocardia*, Nov = *Novosphingobium*, Ped = *Pedosphaeraceae*, Per = *Perlucidibaca*, Pl = *Plesiomonas*, Ps = *Pseudomonas*, Rh = *Rhodobacteraceae*, Sap = *Saprospiraceae*, Spp = *Sphingopyxis*, Spm = *Sphingomonadaceae*, Sta = *Staphylococcus*, Vib = *Vibrio* (**A**) and principal coordinate analysis plots of those samples and tank water, based on Bray–Curtis (**B**) and Jaccard (**C**) similarities. ASV = amplicon sequence variant, TP = time-point, color-bar and symbol legends at bottom; taxonomic abbreviations listed at beginning of manuscript. *p* and F values associated with main effects of time and sample site based on two-way permutation multivariate analysis of variance (PERMANOVA)

In comparison, samples from the effluent water drained from the tanks, analyzed pre- and post-mechanical (particulate) filtration, and post-carbon filtrate returning to the UV disinfection unit revealed apparent similarities to the tank water, particularly at later time-points. While these sites mirrored the composition of the tank water with high relative abundance of *Staphylococcus* and *Cutibacterium* spp., the FBB and post-UV disinfection water samples demonstrated certain initial similarities with regard to dominant taxa followed by their own unique site-dependent temporal progressions (Fig. 4A). Post-UV disinfection water shifted dominance between various Alphaproteobacteria (e.g., *Sphingopyxis*, *Novosphingobium*, *Bradyrhizobium*), Betaproteobacteria (e.g., *Hydrogenophaga*), Gammaproteobacteria (e.g., *Pseudomonas*), and Actinomycetales (e.g., *Cutibacterium*, *Nocardia*), while the FBB started similarly but then showed a steady and gradual increase in evenness among 10 to 12 dominant taxa. PCoA plots reflected a similar progression as seen in tank water, although samples from the post-UV disinfection water and FBB clustered distinctly from the other sites and showed high intra-site similarity at each successive time-point. However, significant time- and site-dependent differences were detected in community structure (time: *p* < 0.0001, F = 5.2, site: *p* < 0.0001, F = 6.1, Fig. 4B) and composition (time: *p* < 0.0001, F = 3.0, site: *p* < 0.0001, F = 2.2, Fig. 4C). Testing for differences in β-diversity and dispersion according to sample site and time-point using the adonis function in the vegan R package yielded comparable results. In agreement with PERMANOVA, adonis detected significant differences in β-diversity associated with time (*p* < 0.001, F = 9.6) and site (*p* < 0.001, F = 8.1). Comparison of multivariate dispersion between groups also detected a significant difference (*p* < 0.001, F = 12.1). It is worth noting that all sites clustered together at the initial time-point, collected immediately prior to population of the tanks with fish, but began diverging after one week of water circulation through the system, suggesting that the observed progression in community structure over time is largely due to the introduction of zebrafish into the system, as well as the influx of nutrients from feeding them. Line graphs representing sequence numbers in the FBB of dominant taxa, and taxa previously associated with ammonia and nitrite oxidation, suggest weekly log-phase increases beginning almost immediately for *Sphingopyxis*, other unresolved *Sphingomonadaceae*, *Hydrogenophaga*, and *Pirellulaceae*, followed by similar expansions in *Rhodobacteraceae*, *Pedosphaeraceae*, and *Blastocatellaceae* beginning at later time-points (Additional file 2). Notably, *Nitrospira* spp. were less abundant than the aforementioned taxa in the FBB by orders of magnitude, and members of the *Nitrosomonadaceae* family were rare to undetected. Similarly, several unresolved members of the *Nitrososphaeraceae* (likely ammonia-oxidizing archaea) were detected but at extremely low prevalence and read counts.

### Core taxa comprise *Actinobacteria, Firmicutes*, and variable *Proteobacteria*

**Fig. 5:**
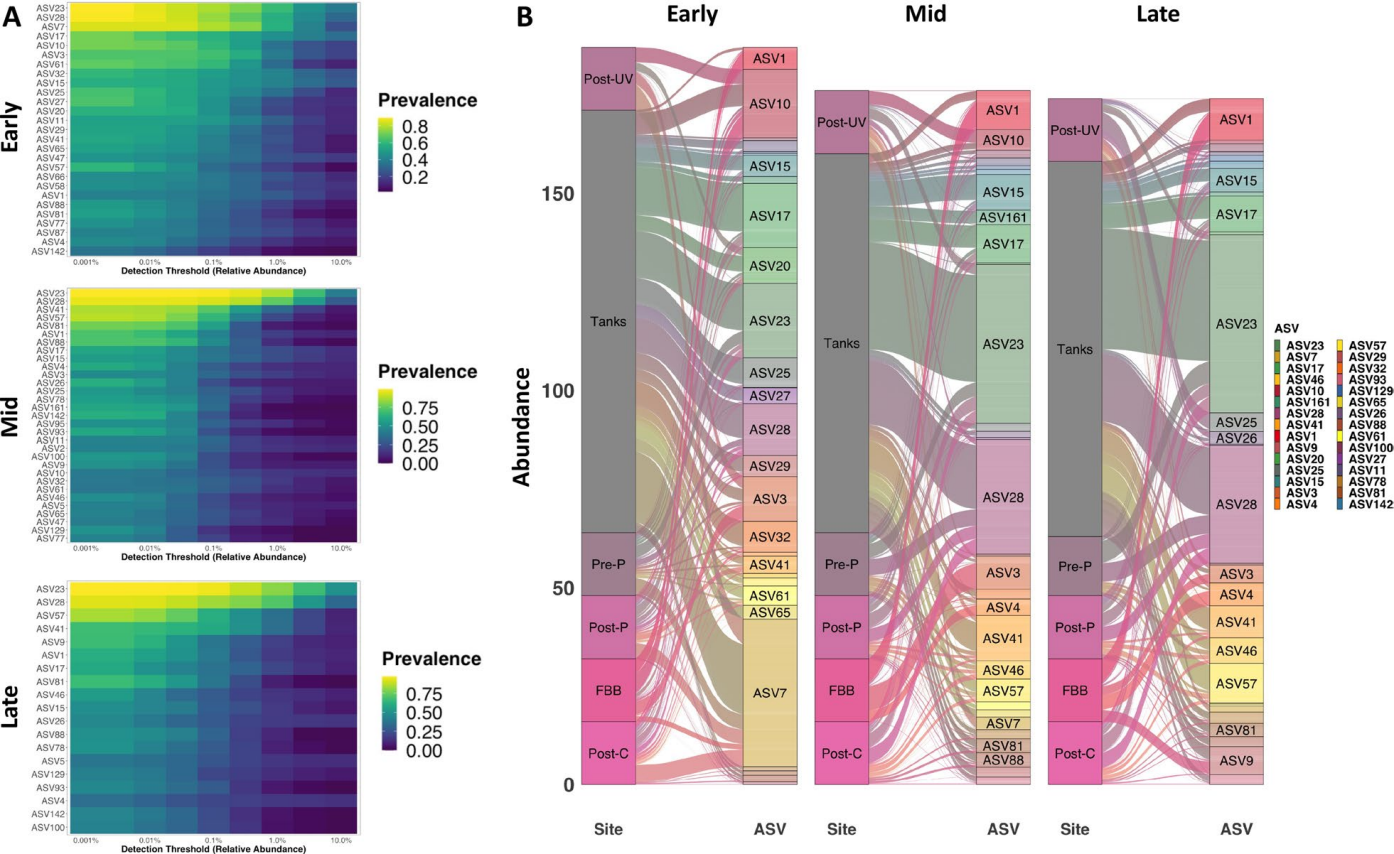
Limited number of taxa comprise core community throughout equilibration. Heatmaps showing prevalence (legend at right) of core ASVs at increasing thresholds of relative abundance at Early (TP1 to TP4), Mid (TP5 to TP8), and Late (TP9 to TP12) time-points (**A**); alluvial plots showing distribution of core taxa among sample sites within each period of time (legend at right) (**B**). See Additional file 3 for taxonomic identity of core ASVs

To define core taxa during establishment of the system microbiome, amplicon sequence variants (ASVs) were stratified by prevalence and relative abundance. To simplify the analysis and interpretation, time-points were divided categorically into early (TP1 to TP4, at 0-3w post-population), mid (TP5 to TP8 at 4-10w post-population), and late (TP9 to TP12 at 12-18w post-population) time-points (Fig. 5A). Within all three periods, ASV23 and ASV28, representing *Cutibacterium* and *Staphylococcus* respectively, were the two dominant taxa system-wide. Other core taxa across all three periods include other members of the Actinobacteria (*Lawsonella*, *Micrococcus*, *Propionibacterium*), *Streptococcus*, *Cetobacterium*, and *Aeromonas* (Additional file 3). While Proteobacteria were commonly identified as core taxa, few were consistently identified as such across all three periods of the study. The distributions of those core taxa within and between each site are shown as alluvial plots (Fig. 5B).

### Microbial network analysis indicates central role for *Nocardiaceae* in FBB

To identify microbial interaction networks associated with the equilibration and stabilization of the tanks, the FBB, and the system as a whole, a graphical lasso regression method was used to infer ecological associations from a sparse matrix such as ASV counts across all time-points. Depending on the site from which the samples were collected, the density of the networks varied. The post-carbon filtration samples had the least density, i.e., connectivity, and were dominated by one or two taxa as indicated by the size of the node. Within each site, smaller clusters were found outside of the largest cluster of taxa indicating the overall niche preferences of the respective taxa and their contributions to the community stability within the system.

**Fig. 6:**
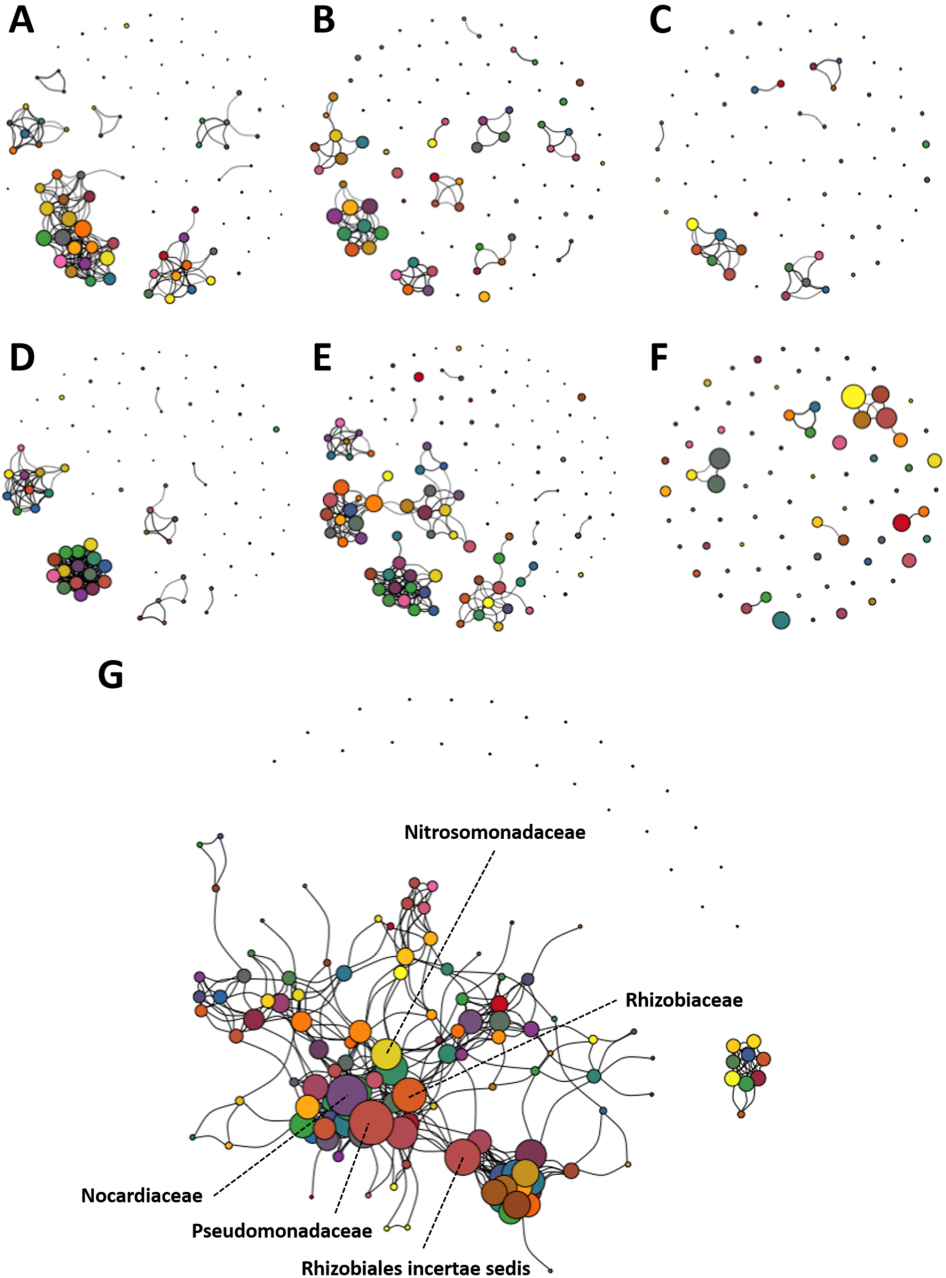
Enriched networks present in FBB and post-UV disinfection water dominated by several *Proteobacteria*. Community network analysis plots based on temporal data for post-UV disinfection unit (**A**), tanks (**B**), pre-particulate filter (**C**), post-particulate filter (**D**), FBB (**E**), post-carbon filter (**F**) samples, and the system as a whole (**G**). Nodes indicate amplicon sequence variants (ASVs) while the edges in the network graphs are weighted based on the relative abundance and indicate the correlation between two nodes. The nodes are colored based on their respective taxonomical classifications and the size of the nodes indicate the highest degree, betweenness, and centrality within the overall network

The predicted interaction networks in tank water, pre- and post-particulate filter water, and post-carbon filter water over time were relatively sparse in contrast to those detected in the post-UV disinfection water and FBB (Fig. 6A through F). The network in FBB identified *Nocardiaceae* as a hub taxon between two sub-networks of bacteria, owing to its high level of degree, betweenness, and centrality. A combined network analysis of the entire system again placed *Nocardiaceae* as pivotal community members alongside *Pseudomonadaceae*, *Nitrosomonadaceae*, Rhizobiales *incertae sedis*, and *Rhizobiaceae* (Fig. 6G).

### Only a fraction of detected ASVs were represented in the culturable fraction

Lastly, to characterize the culturable portion of the system microbiota and aid in identification or resolution of detected taxa, replicates of all samples were serially diluted and plated on four different media selected to grow a broad range of environmental and aquatic bacterial taxa. All cultured isolates were then analyzed via matrix-assisted laser desorption/ionization-time of flight (MALDI-TOF) mass spectrometry and annotated against a protein spectrum database. In total, 269 isolates were recovered, resulting in 33 distinct identifiable isolates (Additional file 4) and three unidentified isolates. The most common isolates were identified as *Pseudomonas alcaliphila*, *Limnobacter thiooxidans*, *Acidovorax facilis*, and *Aeromonas veronii*.

**Fig. 7:**
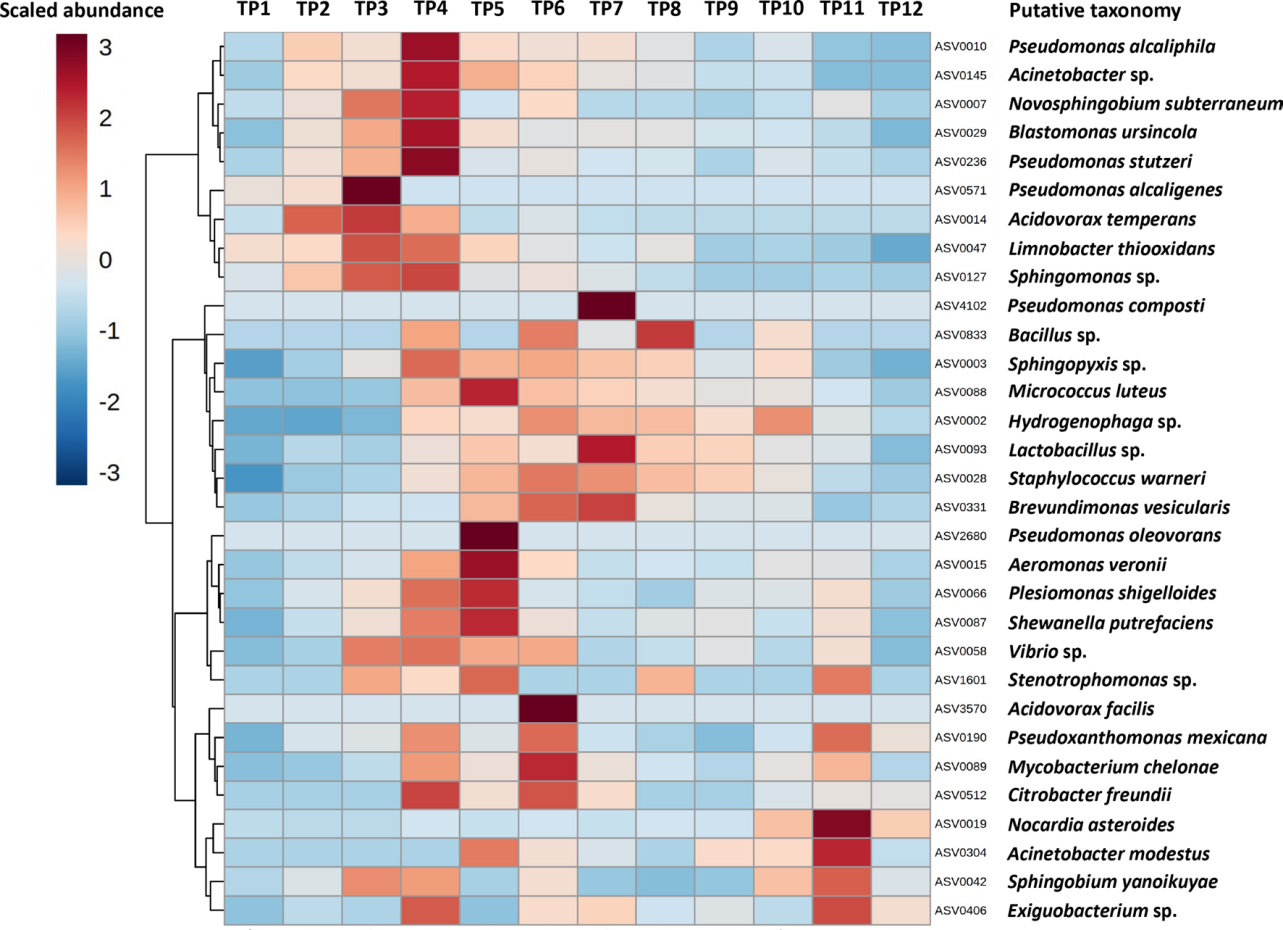
Cultivable taxa expand early during equilibration. Heatmap showing cube root-transformed mean relative abundance (legend upper left) at each time point (TP), of amplicon sequence variants (ASVs) matching the taxonomies assigned to culture isolates via MALDI-TOF

As 16S rRNA amplicon sequencing is unable to resolve certain species or genera (due to genetic homogeneity within a given family at the 16S region in question or lack of relevant taxonomies in the database used for annotation), efforts were made to identify culture isolates within the sequencing data via post hoc comparison of poorly resolved ASV sequences against annotations made using the MALDI-TOF mass spectrometry, with the ultimate goal of visualizing the relative abundance of cultured isolates throughout the entire time-course dataset. While not specific to the cultured isolates, candidate ASVs with a 100% nucleotide identity to a culture isolate at the species level were identified for 17 of the 33 culture isolates. Of the remaining 16 isolates, curation of all poorly resolved ASVs in the next higher taxonomic division returned a 99.6% nucleotide identity in four isolates, or was only annotated to the level of genus via both MALDI-TOF and 16S sequencing in 11 isolates. Only one isolate, *Tsukamurella* sp., could not be matched to a candidate ASV at any taxonomic level. The mean relative abundance in all samples across time of the 32 putative ASVs matching culture isolates is shown in Fig. 7.

## Discussion

Previous studies have suggested that the gut microbiome assemblage of fishes is determined by a combination of extrinsic (e.g., salinity, trophic level) and intrinsic (e.g., host taxonomy) factors [49]. Research focused on the gut microbiome of zebrafish per se suggests that colonization of the zebrafish gut is not a stochastic, or neutral, process, but rather is influenced by active processes including microbe-microbe interactions or host selection [50]. Indeed, Roeselers et al. found evidence of the historical connections between research facilities in the gut microbiome of the fish at those institutions, indicating that zebrafish acquire facility-dependent features within their microbiome over time [51]. While several studies have been performed applying molecular approaches to characterize the microbial community of commercial recirculating aquaculture systems (RAS), we were unable to identify any meaningful surveys of commercial zebrafish housing systems using culture-independent methods. As we were preparing to install and implement a commercial zebrafish housing system for research purposes, we wanted to determine the time point at which the environmental microbiome of a newly installed system had stabilized and would thus be suitable for use in experimental procedures. Based on multiple metrics of community composition, it appears that the tank water and most of the filter water samples have peaked, plateaued, or reached some sort of stable equilibrium by TP4 (3w post-population) to TP6 (6w post-population). This was true of sample coverage across all sites (as a very rough gauge of relative biomass), community richness and α-diversity, and the dominance of the two core taxa, *Staphylococcus* and *Cutibacterium*. That is not to say that no change occurs after TP6 as the PCoA plots demonstrated continued drift in β-diversity throughout the entire study duration. Moreover, the FBB and post-UV disinfection samples showed very different patterns throughout the study period, including different community members and kinetics. This is not surprising with the FBB as this is a fluidized fine sand media designed to facilitate bacterial colonization via high surface area, and inoculated with a proprietary mixture of bacteria at the commercial production facility. However, the presence of a distinct microbiome within the effluent from the UV disinfection unit, completely different from the post-carbon filter water entering the UV disinfection unit, was unexpected. For those samples, the small piece of tubing carrying water from the UV disinfection unit to the tanks was temporarily disconnected from the tank and allowed to drain 1 mL of water directly into a sterile container for DNA extraction. The only difference between this and the post-carbon filter water samples is passage through the UV disinfection unit, yet the communities are strikingly different. The ultraviolet exposure achieved in RAS is inadequate to sterilize water, and bacterial taxa vary widely in their susceptibility to ultraviolet irradiation [52, 53]. The disproportionately high coverage of post-UV disinfection water samples at almost every time point (and clear community progression over time) suggests the presence of a stable biofilm upstream of our sampling site. Ultraviolet disinfection is more effective at reducing individual planktonic bacteria suspended in the water column than for bacterial species that aggregate in water [54], or bacteria incorporated into biofilms that are thus poorly penetrated by ultraviolet radiation. Many members of Actinomycetales are hydrophobic, including *Nocardia* spp. [55], an abundant taxon in the post-UV disinfection water samples, likely reflecting biofilms that became established as the system equilibrated. Alternatively, the bacterial DNA collected from the UV effluent may simply represent a UV-resistant fraction of the community, with reduced fragmentation of the 16S rRNA gene, and subsequent over-representation in the pool of amplicon libraries. These possible explanations are not mutually exclusive.

The high relative abundance of *Staphylococcus* spp. and *Cutibacterium* spp. in tank water and other sites is noteworthy due to the role of each genus as dominant members of the human skin microbiome [56]. Moreover, multiple members of each genus are capable of biofilm formation [57–61], suggesting they might be particularly well-suited for colonization of RAS following dissemination from the skin of individuals maintaining the system.

That the tank water in the new system never became similar in composition to the tank water from the existing housing system was not entirely unexpected. The older system was not commercially purchased, but rather, was constructed from the necessary material according to a published design [27]. As such, tank size, flow-through rate, and filtration systems all varied between system. Additionally, the existing system had been in use for several years, much longer than the study period for the new system.

Regarding the community within the FBB ostensibly responsible for nitrification of the system via ammonia-oxidizing bacteria (AOB), *Nitrosomonas* sp. and related taxa were surprisingly rare, being detected in less than 10% of samples and at extremely low relative abundance, and *Nitrobacter* spp. were not detected at all. In contrast, *Nitrospira* sp. were present at modest relative abundance, primarily in the FBB. *Nitrospira* spp. are known to oxidize nitrites in freshwater aquaria [20], and some species are capable of complete nitrification from ammonia to nitrate [62]. Multiple species of *Spingomonas* and *Sphingopyxis* also express nitrate reduction machinery [63–66], and thus may also play a role in denitrification in the FBB system. In contrast, we note that other dominant taxa identified in the FBB including *Hydrogenophaga*, *Sphingopyxis*, *Pirellula*, and unresolved members of the families *Rhodobacteraceae* and *Sphingomonadaceae* are capable of aerobic denitrification. While we were unable to identify any substantive published reports of the microbiome present in RAS used for research purposes, several recent surveys of the water or bioreactors in commercial RAS used for production of fish or shrimp also identified *Rhodobacteraceae* and Planctomycetes as dominant taxa based on relative abundance of 16S rRNA sequences [67–70]. Similarly, multiple studies have implicated *Hydrogenophaga* as a participant in aerobic denitrification in various closed bioreactor systems [71–73]. One limitation of the current study is that nitrogenous compounds were at or below the lower limit of detection, but the lack of any fish mortality during (or after) the study period suggests that nitrification was occurring. The bacteria traditionally associated with nitrification (e.g., *Nitrosomonas*, *Nitrobacter*) are extremely slow-growing bacteria with a doubling time of 18–70 hours [74–76], with evidence that AOB have a faster generation time than nitrite-oxidizing bacteria (NOB) [75]. Thus, if only these species contributed to denitrification, bacterial reproductive capacity could be a rate-limiting factor in equilibration regardless of how quickly new fish are added. However, in part because the number of prokaryotic taxa that contribute to nitrification is likely larger, it is unclear whether the rate of compositional change would have been different if significantly more fish had been introduced during the initial population of tanks.

Culture-based screening of the system complemented the molecular analysis by demonstrating viability of several core members of the system microbiome, and improving the taxonomic resolution of several of these members. MALDI-TOF mass spectrometry annotations improved the taxonomic resolution relative to putative matches in the 16S rRNA sequencing data in 19 of 33 isolates. The greatest overlap between the core taxa and culturable fraction of the system microbiome was Proteobacteria. Of the 36 taxa defined as core taxa by our criteria (in at least one period of time), only 12 were ostensibly cultured. Of those 12, 10 were *Proteobacteria*, the only non-*Proteobacteria* isolates being *Micrococcus luteus* and *Staphylococcus warneri*. This is likely, at least partially, due to the aerobic culture approach. However, lack of other requirements in the culture media for growth of certain core taxa is also likely.

Four culture media, including two permissive (non-selective) media and two selective media were used to isolate a broad array of bacteria. In order to document changing bacterial communities at timepoints occurring before and after the eutrophication associated with the introduction of live zebrafish and zebrafish feed into the system, non-selective media were selected that would facilitate isolation of bacteria that thrive under both oligotrophic and eutrophic nutrient conditions. The BBL™ Trypticase™ Soy Agar with 5% sheep blood (blood agar) was chosen as an enriched differential and non-selective media. Tryptone Yeast Extract Salts (TYES) Agar is a non-selective minimal medium that facilitates the isolation of oligotrophic and fastidious aquatic microorganisms that either will not grow on enriched media or grow very slowly on enriched media, and are thus easily overgrown by rapidly growing bacterial species. Xylose Lysine Deoxycholate (XLD) Agar is a culture medium that selects for a subset of Gram-negative bacteria and is highly differential, aiding in distinguishing between Gram-negative bacteria with similar colony morphologies. Sheep Blood Agar with Phenylethyl Alcohol (PEA) is a selective medium that inhibits Gram-negative bacteria and thus facilitates isolation of Gram-positive bacterial species.

The temporal network data for individual sites revealed that the post-UV disinfection water and FBB substrate showed the highest co-occurrence and dense patterns among all the samples. The other sites, including the tank water and particulate filtration samples demonstrated fewer interactions. It is plausible that these organisms form a multi-cellular biofilm that may be recalcitrant to extreme environments [77]. Interestingly, the overall system network identified *Nocardiaceae* as a key player in these co-occurring taxa communities in conjunction with *Pseudomonadaceae*. More work including the use of metagenomics and stable-isotope probing experiments will be needed in the future to delineate the exact mechanisms by which two organisms interact. Additionally, it has been shown that in a nitrifying medium or a nitrate-rich environment, *Nitrosomonadaceae* and *Pseudomonadaceae* are thought to be prevalent and mutualistic, especially in biofilms [78, 79]. This may be a plausible reason for the co-occurrence of these taxa, and also for their placement as dominant members of the overall network community. Moreover, Keshvardoust et al*.* demonstrated that enriching the glucose content of media leads to a shift in dominance from *Nitrosomonadaceae* to *Pseudomonadaceae*, indicating a potentially competitive relationship between these taxa within the network. In either case, supplementation, and carefully constructed synthetic community models starting with combinations of specific taxa will be required to validate these findings in the future.

In summary, the data reported here provide a detailed and comprehensive characterization of the prokaryotic communities present at different sites of a research zebrafish RAS during establishment. Collectively, these data suggest that a peak population density occurs at roughly 3 to 4 weeks post-population, although the FBB continued to undergo subtle changes in evenness throughout the 18-week study duration. Moreover, our data strongly suggest the presence of bacterial biofilm communities associated with the UV disinfection unit, representing an unappreciated nidus of bacteria within RAS. Lastly, these data demonstrate the complementary abilities of molecular approaches and traditional culture coupled to MALDI-TOF, to characterize complex microbial communities.

## Supplementary Information

**Additional file 1.** Dot plot showing the number of 16S rRNA amplicon sequences recovered from each sample site (legend at right) at each time-point. Pre-P and Post-P = pre- and post-particulate filter water, FBB = Fluidized bed biofilter substrate, Post-C = post-carbon filter water, TP = time-point. p and F values associated with main effects of time and sample site based on two-way analysis of variance (ANOVA).

**Additional file 2.** Line chart showing mean (± SD) sequence number across time in the fluidized bed biofilter (FBB), of dominant taxa and taxa recognized to participate in the oxidation of ammonia and nitrites, or reduction of nitrates and nitrites, on a Log-scale. Sphingomonadaceae NR includes all sequences matched to that family but not resolved to the level of genus, TP = time-point.

**Additional file 3.** Core taxa found at a minimum of 0.01% mean relative abundance in 50% of all samples during the Early (TP1 to TP4), Mid (TP5 to TP8), or Late (TP9 to TP12) time-points.

**Additional file 4.** Taxonomic identity of 33 different culture isolates listed by time-point at which isolates were cultured (columns TP1-TP3, TP5-TP12), and the sample site (numbers: 1 = post-UV, 2 = tanks, 3 = pre-particulate, 4 = post-particulate, 5 = FBB, 6 = post-carbon). Column labeled ASV_ID and Taxonomic annotation indicate putative matches in the sequencing data, following post hoc BLAST analysis.

## Data Availability

The dataset supporting the conclusions of this article are available in the NCBI Sequence Read Archive (SRA) under BioProject ID: PRJNA674483 (Submission ID: SUB8465482 and SUB8465542).

## Abbreviations

Aer: *Aeromonas*
Al: *Alloiococcus*
Aq: *Aquabacterium*
ASV: Amplicon sequence variant
Bl: *Blastocatellaceae*
Br: *Bradyrhizobium*
Bu: *Burkholderiaceae*
Cet: *Cetobacterium*
Ch: *Chitinophagales*
Cut: *Cutibacterium*
FBB: Fluidized bed biofilter
Fer: *Ferruginibacter*
GM: Gut microbiota
Hy: *Hydrogenophaga*
Lim: *Limnobacter*
Noc: *Nocardia*
Nov: *Novosphingobium*
Ped: *Pedosphaeraceae*
Per: *Perlucidibaca*
Pl: *Plesiomonas*
Ps: *Pseudomonas*
RAS: Recirculating aquaculture system
Rh: *Rhodobacteraceae*
Ru: *Runella*
Sap: *Saprospiraceae*
Spp: *Sphingopyxis*
Spm: *Sphingomonadaceae*
Sta: *Staphylococcus*
UV: Ultra-violet
Vib: *Vibrio*
