# Supplementary figures and images for: Molecular and culture-based assessment of the microbiome in a zebrafish (Danio rerio) housing system during set-up and equilibration
Source: Anim Microbiome. 2021 Aug 5;3:55. doi: 10.1186/s42523-021-00116-1 (PMC8340428; doi:10.1186/s42523-021-00116-1)

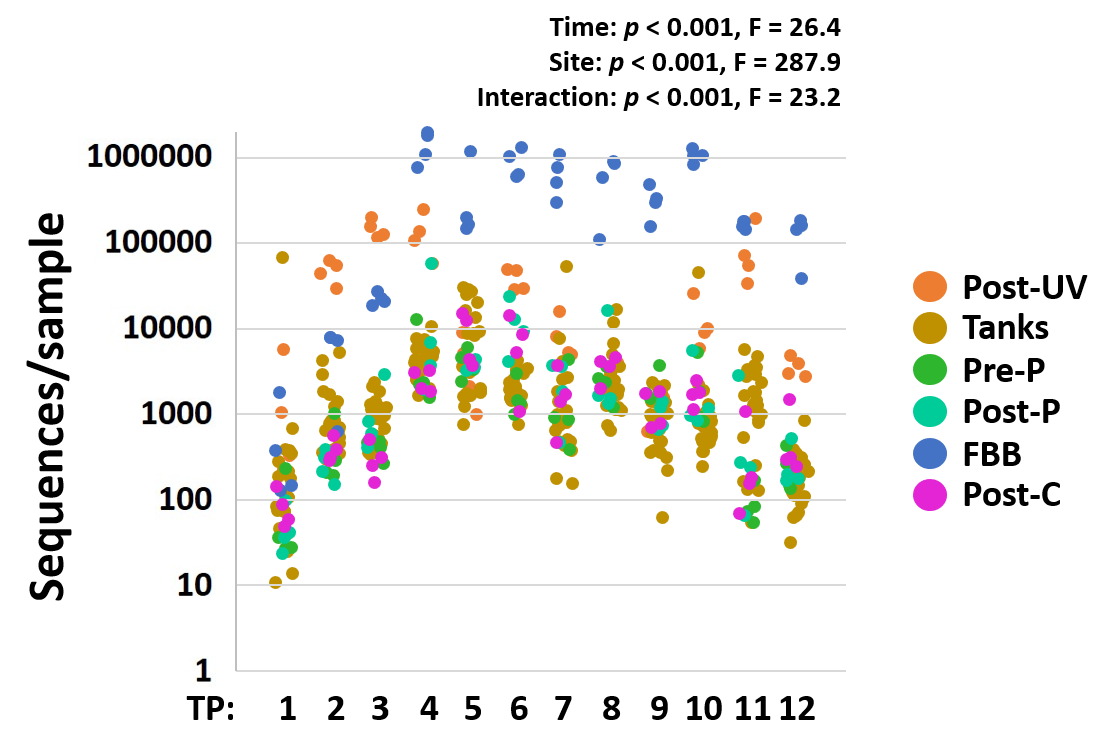

Supplement: Supplementary file 1 — Additional file 1. Dot plot showing the number of 16S rRNA amplicon sequences recovered from each sample site (legend at right) at each time-point. Pre-P and Post-P = pre- and post-particulate filter water, FBB = Fluidized bed biofilter substrate, Post-C = post-carbon filter water, TP = time-point. p and F values associated with main effects of time and sample site based on two-way analysis of variance (ANOVA). [file 42523_2021_116_MOESM1_ESM.tif]

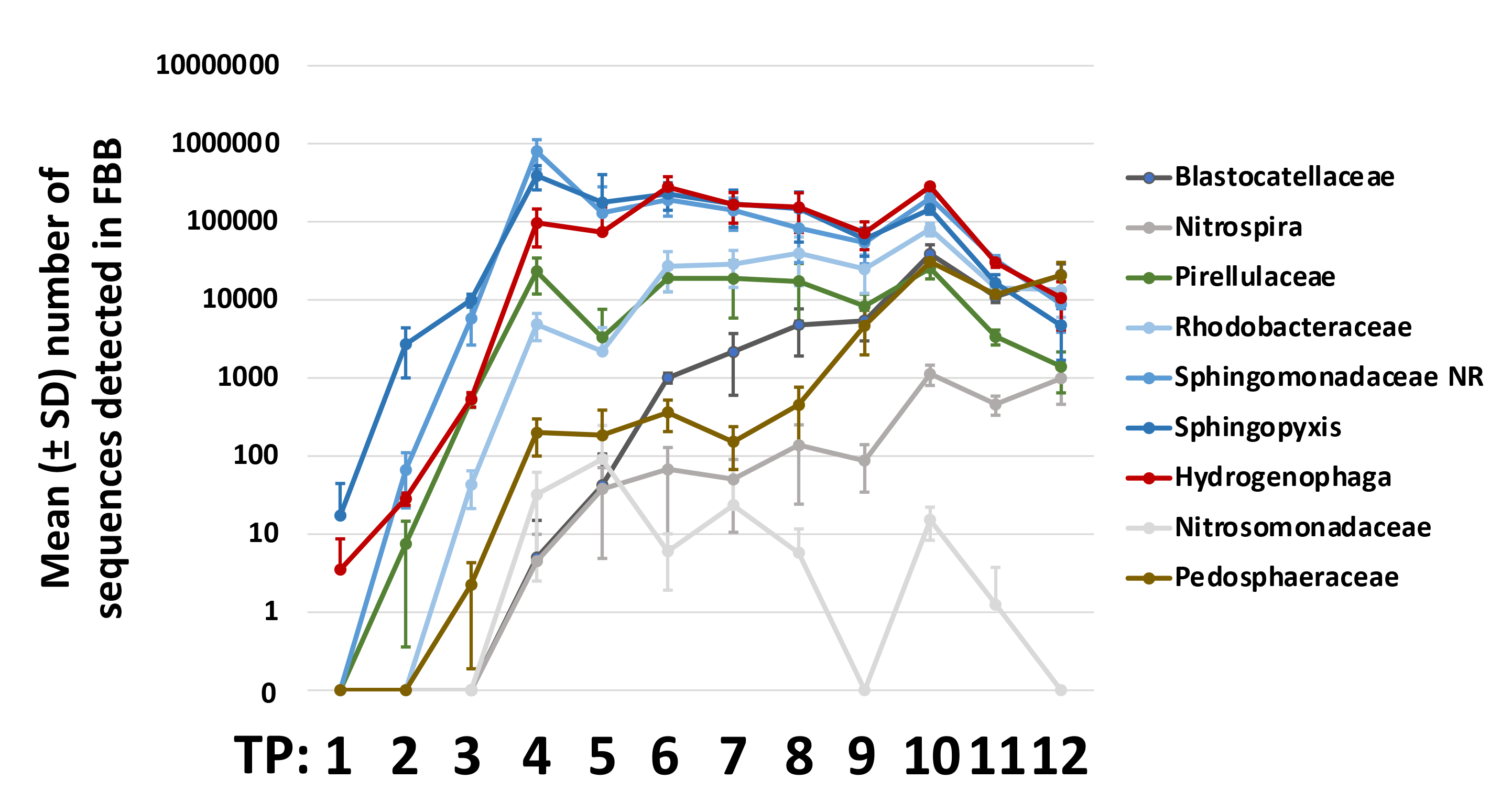

Supplement: Supplementary file 2 — Additional file 2. Line chart showing mean (± SD) sequence number across time in the fluidized bed biofilter (FBB), of dominant taxa and taxa recognized to participate in the oxidation of ammonia and nitrites, or reduction of nitrates and nitrites, on a Log-scale. Sphingomonadaceae NR includes all sequences matched to that family but not resolved to the level of genus, TP = time-point. [file 42523_2021_116_MOESM2_ESM.tif]
